# Genome-wide profiling of chicken dendritic cell response to infectious bursal disease

**DOI:** 10.1186/s12864-016-3157-5

**Published:** 2016-11-05

**Authors:** Jian Lin, Jing Xia, Keyun Zhang, Qian Yang

**Affiliations:** College of Life Science, Nanjing Agricultural University, Weigang 1, Nanjing, Jiangsu 210095 People’s Republic of China

**Keywords:** Infectious bursal disease virus, Dendritic cells, miRNA, lncRNA

## Abstract

**Background:**

Avian infectious bursal disease virus (IBDV) is a highly contagious, immunosuppressive disease of young chickens, which causes high mortality rates and large economic losses in the poultry industry. Dendritic cells (DCs), which are antigen-presenting cells, have the unique ability to induce both innate and acquired immune responses and may significantly influence virus pathogenicity. To understand the interaction between IBDV and DCs, a microarray was used to analyse the response of DCs infected by IBDV.

**Results:**

IBDV infection induced 479 upregulated and 466 downregulated mRNAs in chicken DCs. Analysis of Gene Ontology suggested that transcription from the RNA polymerase II promoter and the RNA biosynthetic process were enriched, and pathway analyses suggested that oxidative phosphorylation, as well as the T cell receptor and Interleukin-17 (IL-17) signalling pathways might be activated by IBDV infection. Moreover, microRNA (miRNA) and long non-coding RNA (lncRNA) alterations in IBDV-infected chicken DCs were observed. A total of 18 significantly upregulated or downregulated miRNAs and 441 significantly upregulated or downregulated lncRNAs were identified in IBDV-stimulated DCs. We constructed 42 transcription factor (TF)–miRNA–mRNA interactions involving 1 TF, 3 miRNAs, and 42 mRNAs in IBDV-stimulated DCs. Finally, we predicted the target genes of differentially expressed lncRNAs, and constructed lncRNA-mRNA regulatory networks.

**Conclusions:**

The results of this study suggest a mechanism to explain how IBDV infection triggers an effective immune response in chicken DCs.

**Electronic supplementary material:**

The online version of this article (doi:10.1186/s12864-016-3157-5) contains supplementary material, which is available to authorized users.

## Background

Infectious bursal disease, induced by infectious bursal disease virus (IBDV), is a highly contagious, immunosuppressive disease of young chickens, which causes high mortality rates and large economic losses [1, 2]. IBDV is a member of the genus Avibirnavirus of the family Birnaviridae, and has bi-segmented double-stranded RNA. IBDV infection damages the precursors of antibody-producing B lymphocytes in the bursa of Fabricius and causes severe immunosuppression and mortality in young chickens [3, 4]. Vaccination against IBDV has been a viable option for controlling IBDV for many years [5]. However, this virus is still prevalent, which may be attributable to its horizontal transmission and the reversion to virulence of live IBDV vaccines [6–8]. Thus, controlling IBDV is a major challenge in poultry health management.

Understanding the immune mechanisms underlying protection against IBDV is important for preventing infection. Recent studies have focused on the molecular mechanisms involved in host responses upon IBDV infection [9]. Microarray assays and proteomic approaches have been employed to reveal the molecular mechanisms of host cells in response to IBDV infection [10, 11]. The majority of these studies emphasised the responses of DF-1 chicken embryo fibroblasts or chicken tissues. Thus, there is limited information regarding the function of dendritic cells (DCs) during IBDV infection. DCs are antigen-presenting cells with the unique ability to induce both innate and acquired immune responses [12]. Chicken DCs were originally identified in primary and secondary lymphoid organs, and are composed of several subtypes including bursal secretory dendritic cells (BSDCs), follicular dendritic cells (FDCs), and thymic dendritic cells (TDCs) [13, 14]. Our previous study found that inactivated IBDV stimulation significantly elevated chicken DC surface markers and enhanced the ability to induce the T-cell proliferative response, whereas IBDV infection only slightly upregulated CD40 and CD86 compared to marked increases in CD40 and CD86 in LPS-induced and inactivated IBDV groups [15]. These data indicate that IBDV may escape the recognition of chicken DCs. Moreover, we found that IBDV-stimulated chicken DCs did not show any stimulating effect in terms of activating lymphocytes. The different capacities of viruses to efficiently stimulate DC maturation and antigen presentation account for their different levels of immunogenicity. In this regard, the lower maturation of DCs induced by IBDV might contribute, at least in part, to the poor ability of the vaccine to produce immunity. These results reveal that IBDV infection impairs DC maturation and function, which may explain why chickens infected with this virus fail to trigger effective specific immune responses or develop immune memories.

Non-coding RNAs have emerged as key regulators of diverse biological processes, including innate immune responses [16]. For example, microRNAs (miRNAs) influence the development of DCs and their ability to present antigens, as well as viral replication, whereas long non-coding RNAs (lncRNAs) may play roles in regulating the defence against viruses [17–19]. To further explore the potential roles of miRNAs and lncRNAs in IBDV infection, microarray and bioinformatic analyses were employed to reveal the mechanism underlying IBDV infection of DCs. The results of this study may provide clues for novel preventive or therapeutic strategies against IBDV infection and accelerate vaccine development.

## Methods

### Animals and viruses

Four to six-week-old inbred line ROSS 308 chickens were kindly provided by the Jiangsu Academy of Agricultural Sciences (JAAS) (Nanjing, China). All animals were maintained at an animal facility under pathogen free conditions. A tissue culture infectious dose 50 of 10^−6.38^/0.1 ml of the IBDV strain (B87; JAAS, China) was used.

### Culture of chicken BMDCs and its identification

Chicken BMDCs were generated and expanded in vitro by culturing bone marrow precursors with 50 ng/ml recombinant chicken granulocyte colony-stimulating factor (GM-CSF, Abcam, USA) and 50 ng/ml IL-4(Kingfisher, USA), as described [20, 21]. At day 6, the non-adherent, relatively immature DCs were harvested and placed in fresh medium (1 × 10^6^ cells/ml) with LPS (1 μg/ml) stimulation. Cells were then collected, washed and incubated at 4 °C for 30 min with PE-conjugated anti-human CD11c (0.05 mg/ml), FITC-labeled anti-chicken major histocompatibility complex class II (MHC-II) antibody (0.5 mg/ml). After three times washing, cells were analyzed with Fluorescence Activated Cell Sorter (FACS) (BD, FACS Aria) (Additional file 1). Cells were incubated with IBDV at a multiplicity of infection of 1 (MOI = 1) for 12 h.

### Microarray experiments

Cultured chicken BMDCs were randomly divided into either control or IBDV-stimulated groups. Each group consisted of three wells of BMDCs from three chickens. Total RNA and miRNA were separately isolated using the RNeasy Total RNA Isolation Kit (QIAGEN, Germany). A chicken microarray was purchased from RiboBio, and the microarray (containing mRNA and lncRNA, RiboArray™ Custom Array (A10000-1-90) ) was hybridised. Raw data were normalised using the RMA method [22]. Samples were labeled with ULS-Cy5. Labelling efficiency was calculated by the concentration of CyDye and RNA, as measured by spectrophotometry. Imaging was performed using a laser scanner GenePix4000B (Molecular Device). Normalised intensities were transformed into gene expression log2 values. The ratio and log2 ratio of normalised spot intensities between the test and control samples were calculated. *P* values were calculated using the analysis of variance (ANOVA) method.

### The identification and bioinformatics analyses of different expressed mRNAs

Differentially expressed mRNAs between control chicken DC group and IBDV stimulated group were determined with a cut off of at least 2-fold change and a P value less than 0.01. Such genes were subject to GO categorization, KEGG (Kyoto Encyclopedia of Genes and Genomes) and BIOCARTA pathway analyses. Analyses were performed with DAVID (the Database for Annotation, Visualization and Integrated Discovery) by using an independent list of differentially-expressed genes.

### The identification of different expressed miRNAs and its target prediction, GO categorization, and pathway analyses

Differentially expressed miRNAs were chosen with a cutoff of at least 2-fold change and a *p* value less than 0.05. Potential targets of these miRNAs were predicted using the software miRDB and TargetScan. Taking the intersection of the two predictions, we obtain the optimal potential target. MiRNAs in which no potential target was predicted using two software. MiRDB or TargetScan software was individually used to predict potential targets. To further understand the potential functions of miRNA-target genes, GO categorization and pathway analysis were assigned using the DAVID gene annotation tool.

### The identification of different expressed lncRNAs and its association analyses with different expressed mRNA

Differentially expressed lncRNAs were first chosen with a cutoff of at least 2-fold change and a *P* value less than 0.01. Since transcriptional regulation by lncRNAs could work either in cis or in trans, We then predicted the cis and trans target gene of the difference expressed lncRNA. For the cis target genes, we mapped the genomic location of lncRNA and those proteins encoding mRNA. We predicted and selected four type lncRNA cis targets, including the sense exon, antisense exon and sense intronic, as well as those 1 kb away from the initial position (bi-direction). For the trans target, we initially extracting the 3′ UTR sequence of different expressed lncRNA and mRNA, then blasted the lncRNA and searched for the complementary region of the three prime untranslated region (3′UTR). After searching, we use RNAplex, a software which can predict the stability and binding ability of the complex formed by lncRNA and 3′ UTR, to further narrow the range of cis target genes by setting the main parameters free energy of RNAplex < −100. Furthermore, we calculated the Pearson Correlation Coefficient to predict the lncRNA’s co-expression target genes. The calculation formula is as follows and we selected the target genes according to Pearson correlation coefficient absolute value range of 0.9 and significant correlation with *P* value of 0.01. Finally, we constructed the co-expression regulatory network, visualized by cytoscape, based on predicting target genes and different expressed lncRNA.

### QRT-PCR confirmation of mRNA, miRNA and lncRNA microarray results

Based on the microarray results, 22 mRNAs, 10 miRNAs and 7 lncRNAs were selected and examined by qRT-PCR. For real-time PCR, 7500 Real-Time PCR System (ABI) and SYBR Green Master (Takara) were used. Each sample and negative controls had at least three technical replicates. Gene-specific primers for mRNAs, miRNAs and lncRNAs were separate listed in Tables 1, 2 and 3. GAPDH, β-actin and 5S rRNA were amplified under the same conditions as internal controls (Additional file 2). The relatives fold change was calculated based on the -ΔΔct method [23].

**Table 1:** QRT-PCR primers used in verification of avian mRNAs results

Gene	Sence	Anti-sence	Product
GAPDH	TGACCACTGTCCATGCCATC	CAGCAGCCTTCACTACCCTC	273 bp
β-ACTIN	tgtatgccaacacagtgctg	cctgcttgctgatccacatc	204 bp
MAPK Signaling Pathway			
EGF	gtactggtgcgatgctaagc	tgttttggccggtcttcttg	185 bp
PIK3CB	cacggtcgcatttggatcat	atccactgcccagatgtcaa	202 bp
MAP4K4	cccattccttcagtgaccct	actggtgatcgggatgttgt	208 bp
FGF1	ggcttgtaccaaagcagacc	gctgggctctcactcagtaa	240 bp
STAT1	ttaacgaggagctggtggag	gaaaagactgtgcgttcggt	233 bp
SOD1	taccggcttgtctgatggag	tcctccctttgcagtcacat	172 bp
PDGFB	tgaacccggcatgaatttcg	tctcgagacagggacacatg	234 bp
ELK4	agagcccaaagagcaggatt	ggtcagcaagatgggagtct	205 bp
SPRED2	atcctgatgacgtggactcc	accccgtttgttctcctcat	230 bp
TRAF2	ccgttcctgctgattgagtg	cgcagccatcacaagtcaat	190 bp
NFATC2	aactattgactgtgcgggga	ctcgatggggtttgatgctg	172 bp
MAP4K3	tcagggtggctacttcttgg	tggtaaaggaggtggcactt	163 bp
EGFR	ttggagaagggagagcgttt	ccatgtcctcctcctccatc	244 bp
JAK-STAT signaling pathway			
PIK3CB	cacggtcgcatttggatcat	atccactgcccagatgtcaa	202 bp
SOCS3	accactacatgcctcccac	cgttgacagtcttacggcag	174 bp
CREBBP	ttcccagcaactaggaccag	gccactttcctcttcctcct	243 bp
TPO	gggccaatctgcgacaatag	cacgccgcttaatctcatcc	175 bp
CCND3	tttctggatgctggaggtgt	atgcagagcttctccacagt	195 bp
IL15	gccggagagtcagaaaacac	agtgatttgcttctgtctttggt	155 bp
SPRED2	agaacaaacggggtcaatgc	gcggcaagtaacagcacata	231 bp
OSMR	tcaagctgaagtgtggcaac	cgctccatctccagaaatgc	161 bp
IFNGR1	tggagtcggcagaagatgtt	gaaaccactggacctgagga	217 bp

**Table 2:** QRT-PCR primers used in verification of avian miRNAs results

MiRNAs	Sence primer
gga-let-7 g-5p	TGGGTGGGTGAGGTAGTAGTTTGTACAG
gga-miR-1603	AGTGGTTGGTTTGGTGGTGTC
gga-miR-1635	TGCCCAGGCTGTGCTGTGCTCTGGG
gga-miR-1a-3p	CGGGTGGAATGTAAAGAAGTATGTAG
gga-miR-1715	CGAGGATCAGTAGAAGTCAGCTGTGC
gga-miR-196-2-3p	CGCTACAGCACGAAACTGCCTTAAG
gga-miR-1644	TCTGTTGTGCAGGGCTGTGCT
gga-miR-1778	GGAAGAGAAATGGTCTGCT
gga-miR-302b-3p	GGTAAGTGCTTCCATGTTTTAGTAG
gga-miR-21-5p	GCGTAGCTTATCAGACTGATGTTGA

**Table 3:** QRT-PCR primers used in verification of avian lncRNAs results

Gene	Sence	Anti-sence
GAPDH	TGACCACTGTCCATGCCATC	CAGCAGCCTTCACTACCCTC
β-ACTIN	tgtatgccaacacagtgctg	cctgcttgctgatccacatc
APLF	ggaagggagatgtctgtcca	gggagcttgttggcttgtag
C12ORF43	ttggtttctgcaaatggaca	tttcccagcatccttcattc
FBXO2	atccctcaggacaatgatgc	atgcagaatccatccaaagc
LANCL3	tgagctcgttcactggtgtc	gcagcaggaacacataagca
MYOZ1	cttggatgccttcaggagag	caaggtgcagtcctttggat
TMEM130	tctttgtcctgctgtgcatc	ggcagcttcttgagatgacc
UBE2QL1	tcacttgagcggatgttgag	tacctgcaatgcactgcttc

### Construction of TF (transcription factor)-miRNA-mRNA regulatory loops

TF-miRNA-mRNA loops, representing putative regulatory mechanisms, were constructed based on the miRDB and ChIPBase databases. We first used ChIPBase to construct TF-miRNA regulatory networks [24]. Considering differentially expressed miRNAs in IBDV stimulated DCs, miRNAs promoter region was defined as the 5 kb upstream to 1 kb downstream region. We also extracted TF and miRNA target gene information from ChIPBase. Finally, we constructed the regulatory networks of TF–miRNA–mRNAs. Interactions were imported to Cytoscape for visualization.

### Statistical analyses

Data were evaluated by unpaired two-tailed Student’s t-test using GraphPad Prism 5 (http://www.graphpad.com/) (CSSN), with *P* values < 0.05 considered to be statistically significant. The significance of the data was also determined by one-way ANOVA, followed by Tukey’s multiple comparison tests. All data are expressed as mean ± standard error of the mean.

## Results

### Identifying differentially expressed mRNAs and further bioinformatic analyses of avian BMDCs stimulated by IBDV

**Fig. 1:**
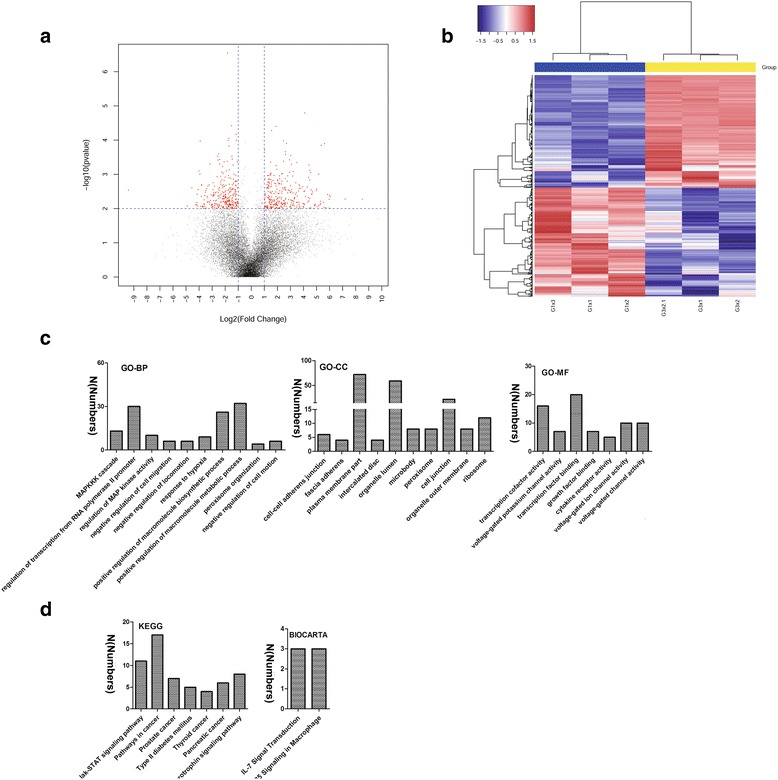
mRNA microarray analyses of IBDV-infected chicken DCs. **a** Volcano plot map of mRNA expression in control and IBDV-infected DCs at 12 h post-infection. A comparison of expression data was performed using an XY-scatter plot analysis of the log base two-fold change. Data points shown in red represent significant differentially expressed genes; *P* < 0.01. **b** Heat map of differentially expressed mRNAs in IBDV-stimulated chicken DCs (The blue one represent control group, which were the cultured DCs, whilst the yellow one represent IBDV-infected DCs. The G1 means group1, which was the control DCs group. While the G3 means group3, which was the IBDV-infected DCs). All of the biological replicates were pooled and calculated to identify differentially expressed mRNAs based on a threshold fold change > 2 and *P* < 0.01. **c** Primary GO categorisation based on differentially expressed mRNAs in IBDV-stimulated chicken DCs. **d** KEGG and BIOCARTA pathway analyses based on differentially expressed mRNAs in IBDV-stimulated chicken DCs

Since our previous study suggested that IBDV stimulation promoted the maturation of avian BMDCs [15], we used microarray analysis to investigate the underlying mechanism. Initially, mRNA expression was examined after avian DCs were stimulated by IBDV. Based on the criteria of at least a 2-fold change and a *P* value less than 0.01, we identified 479 upregulated and 466 downregulated genes in the IBDV-stimulated group (Fig. 1a, b, Table 4 and Additional file 3). Moreover, Gene Ontology (GO) categorisation and pathway analyses of differentially expressed genes were performed using the DAVID online software package. To obtain a better understanding of the potential roles of host factors involved in IBDV infection, GO biological process categories were analysed. MAPKKK cascade, regulation of transcription from the RNA polymerase II promoter, and regulation of MAPK activity were identified for the IBDV-stimulated group (Fig. 1c and Additional file 4). We also performed pathway analyses using the KEGG and BIOCARTA databases. KEGG analysis showed that genes differentially regulated by IBDV, compared with those in control cells, were involved in JAK-STAT signalling, cancer pathways, and type II diabetes mellitus, among others (Fig. 1d and Additional file 4). Using the BIOCARTA database, genes influenced by IBDV were shown to be involved in IL-7 signal transduction and CCR5 signalling in macrophages (Fig. 1d), indicating that IBDV might affect host responses.

**Table 4:** The number of differentially expressed mRNAs, miRNAs and lncRNAs in IBDV treated group

Comparison	Up-regulated	Down-regulated
mRNA	499	466
miRNA	7	11
LncRNA	25	79

### Identifying differentially expressed miRNAs, miRNA target gene prediction, and further bioinformatic analyses of avian BMDCs stimulated by IBDV

**Fig. 2:**
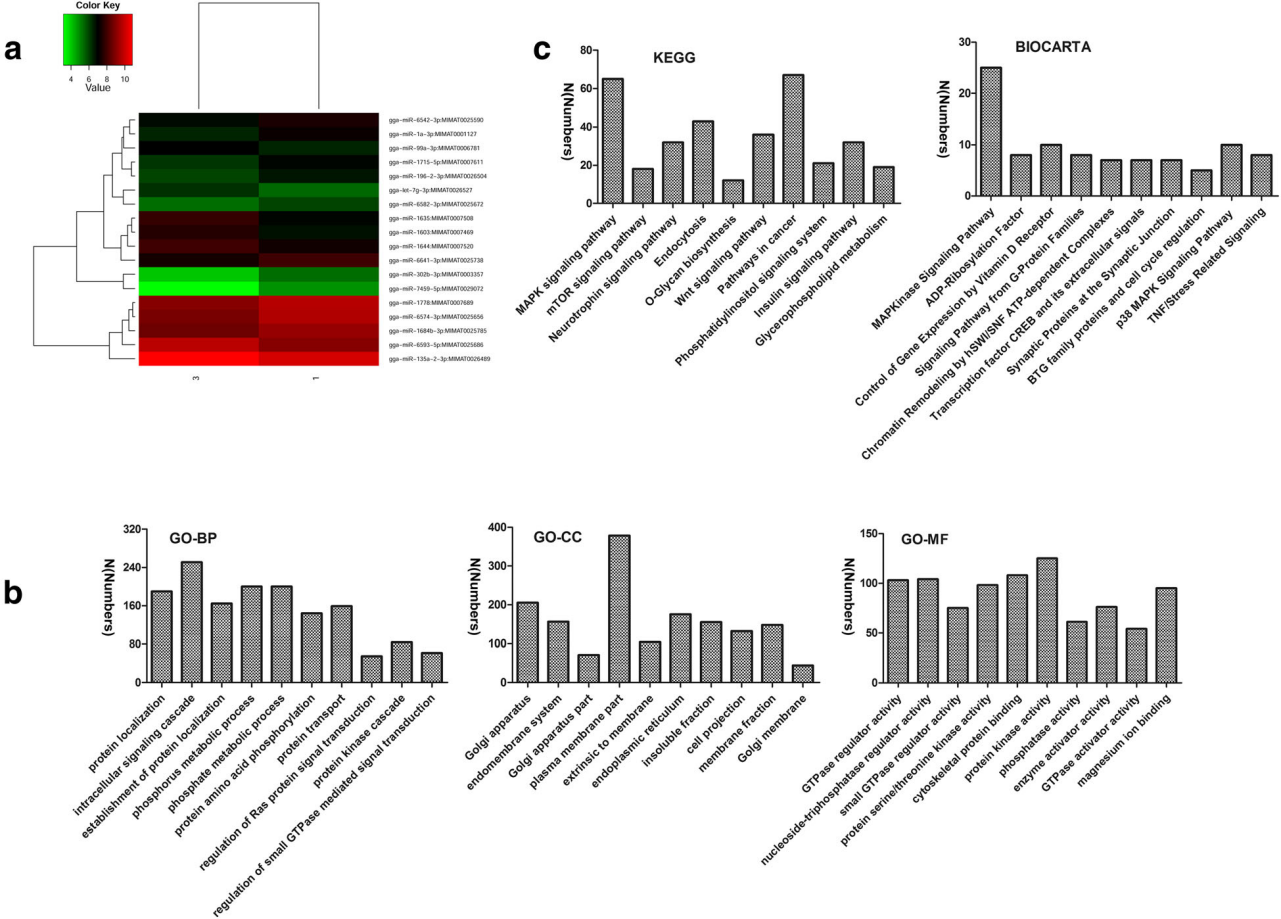
miRNA microarray analyses of IBDV-infected chicken DCs. **a** Heat map of differentially expressed miRNAs in IBDV-stimulated chicken DCs. All of the biological replicates were pooled and calculated to identify differentially expressed mRNAs based on a threshold fold change > 2 and *P* < 0.05. **b** Primary GO categorisation based on target genes from differentially expressed miRNAs in IBDV-stimulated chicken DCs. **c** KEGG and BIOCARTA pathway analyses based on target genes from differentially expressed miRNAs in IBDV-stimulated chicken DCs

miRNAs are key factors regulating the function of DCs in capturing and presenting antigens. Therefore, we studied the influence of IBDV infection on global miRNA expression in chicken DCs. We detected 991 conserved miRNAs and identified 18 miRNAs that were significantly altered by IBDV infection, of which 11 were downregulated and 7 were upregulated (Fig. 2a, Table 4 and Additional file 5). Since miRNAs act by directly silencing or indirectly reducing the expression of their target genes, we next predicted the potential targets of differentially expressed miRNAs using miRDB and TargetScan. Considering only those predicted by both programs, we forecasted 2317 target genes for the differentially expressed miRNAs (Additional file 5). To gain insight into their functions, we performed GO and pathway annotations of the predicted targets using DAVID. In terms of the results of GO term annotation, protein localisation, intracellular signalling cascade, establishment of protein localisation, phosphorus metabolic processes, and phosphate metabolic processes were the top five categories associated with IBDV stimulation (Fig. 2b). The KEGG pathway analysis identified that processes such as MAPK, mTOR, and neurotrophin signalling pathways, as well as the endocytic pathway, were associated with IBDV infection (Fig. 2c and Additional file 6). Using the BIOCARTA database, the primary pathways associated with IBDV infection were shown to involve MAPK signalling, P38 signalling, the transcription factor CREB, and its extracellular signals, among others (Fig. 2c and Additional file 6). The abovementioned results suggest that these differentially expressed miRNAs play crucial roles in response to IBDV stimulation.

### Identifying differentially expressed lncRNAs, lncRNA target gene prediction, and further bioinformatic analyses of avian BMDCs stimulated by IBDV

**Fig. 3:**
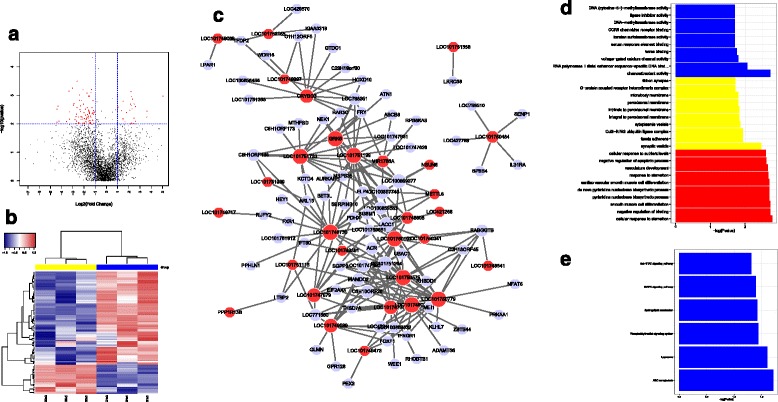
lncRNA microarray analyses of IBDV-infected chicken DCs. **a** Volcano plot map of lncRNA expression in control DCs and IBDV-infected DCs at 12 h post-infection. A comparison of expression data was performed using an XY-scatter plot analysis of the log base 2-fold change. Data points shown in red represent significant differentially expressed genes; *P* < 0.01. **b** Heat map of differentially expressed lncRNAs in IBDV-stimulated chicken DCs. All of the biological replicates were pooled and calculated to identify differentially expressed lncRNAs based on a threshold fold change > 2 and *P* < 0.01. **c** Potential interaction network among the significant differentially expressed lncRNAs (fold change > 2 and *P* < 0.01) and target genes with cytoscape (red represents co-expressed lncRNAs, whereas gray represents co-expressed mRNAs). **d** Primary GO categorisation based on target genes from differentially expressed miRNAs in IBDV-stimulated chicken DCs. **e** KEGG and BIOCARTA pathway analyses based on target genes from differentially expressed miRNAs in IBDV-stimulated chicken DCs

lncRNAs were recently identified as novel regulators of many biology processes, especially lncRNAs involved in regulating mouse DCs. We investigated the influence of IBDV infection on global lncRNA expression in chicken DCs. Based on the criteria described in “The identification of different expressed lncRNAs and its association analyses with different expressed mRNA” section, we identified 25 upregulated and 79 downregulated lncRNAs among a total of 3900 lncRNAs upon stimulation with IBDV (Fig. 3a, b, Table 4 and Additional file 7). Since lncRNAs might exert their effects by *cis*- or *trans*-regulation of target genes, we predicted the potential *cis*- or *trans*-target genes as described in “The identification of different expressed lncRNAs and its association analyses with different expressed mRNA” section. In total, 25 *cis*-targets and 4140 *trans*-targets were identified (Additional file 8). Because lncRNAs may also exert their functions by regulating co-expressed genes, we identified a group of 222 co-expressed lncRNAs/genes, and mapped their interactions (Fig. 3c). To further illustrate the functions of lncRNAs associated with IBDV infection, GO categorisation and pathway analyses of differentially expressed genes were performed with DAVID. Based on the GO analyses, the biological processes active in IBDV-stimulated DCs were concluded to be a cellular response to starvation, negative regulation of binding, and negative regulation of the apoptotic process, among others (Fig. 3d and Additional file 9). In addition, KEGG pathway analyses indicated that the JAK-STAT and MAPK signalling pathways were the main factors associated with IBDV infection (Fig. 3e and Additional file 9).

### Confirmation of microarray results by quantitative reverse transcription PCR

**Fig. 4:**
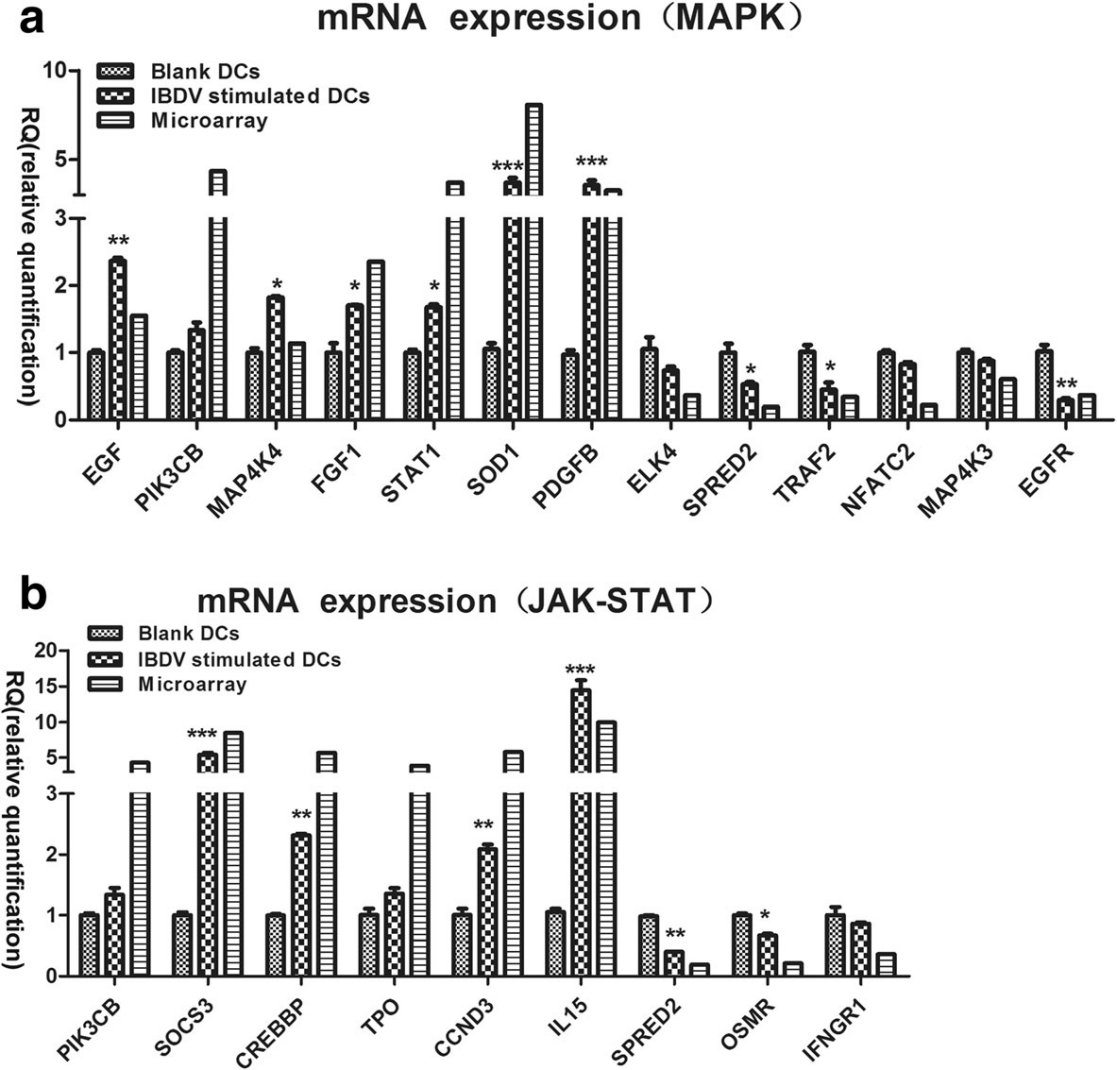
qPCR results of select mRNAs stimulated by IBDV in chicken DCs (All of the experiments were performed at least in triplicate. Significant differences between the treated and control groups are expressed as *P < 0.05 and **P < 0.01, respectively). **a** Expression levels of mRNAs involved in the MAPK signalling pathway. **b** Expression levels of mRNAs involved in the JAK-STAT signalling pathway

**Fig. 5:**
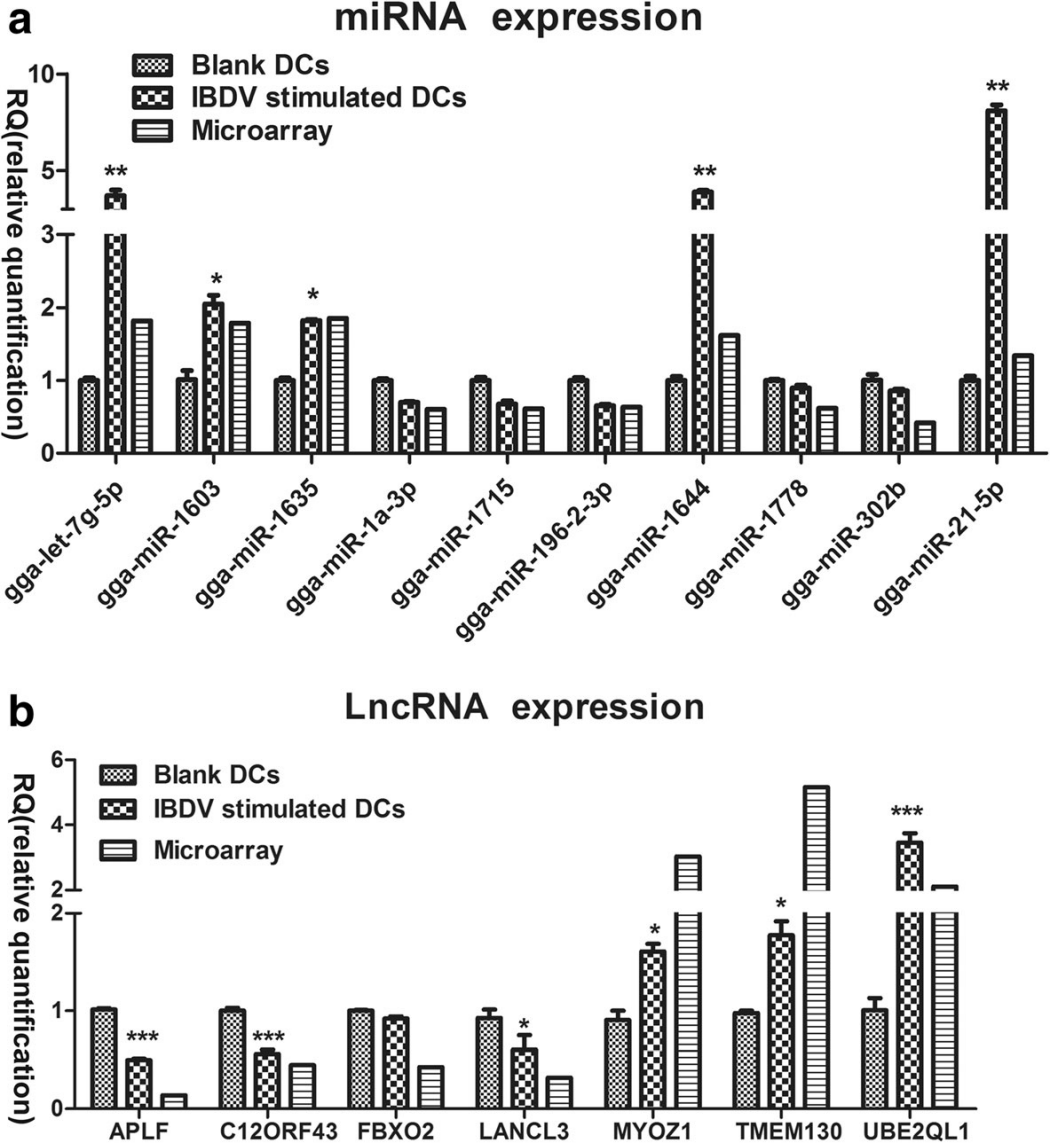
qPCR results of select miRNAs and lncRNAs stimulated by IBDV in chicken DCs (Data are shown as the means ± SD of three samples. **P < 0.01, *P < 0.05 compared with control DCs. Results are representative of three independent experiments). **a** qPCR results of the significantly upregulated or downregulated miRNAs in the IBDV-stimulated group. **b** qPCR results of significantly upregulated or downregulated lncRNAs in the IBDV-stimulated group

To validate the mRNA, miRNA, and lncRNA microarray results, we subjected 22 differentially expressed mRNAs, 10 differentially expressed miRNAs, and 7 differentially expressed lncRNAs to quantitative reverse transcription PCR (qPCR). The PCR data generally matched the microarray data. The fold-change values of representative mRNAs, miRNAs, and lncRNAs in qPCR displayed similar trends as the microarray results. More specifically, the selected mRNAs were mainly associated with the MAPK and the JAK-STAT signalling pathways. For the MAPK signalling pathway, most genes (EGF, *PIK3CB*, *MAP4K4*, *FGF1*, *SOD1*, *STAT1* and *PDGFB*) were significantly upregulated by IBDV, whereas *ELK4*, *TRAF2*, *MAP4K3*, *NFATC2*, *EGFR* and *SPRED2* were downregulated (Fig. 4a). For the JAK-STAT signalling pathway, the expression levels of *CCND3*, *SOCS3*, *TPO* and *IL15* were significantly increased by IBDV stimulation, whereas those of *SPRED2*, *IFNGR1* and *OSMR* were decreased (Fig. 4b). In addition, of the 10 tested miRNAs, those with significantly increased levels due to IBDV stimulation were gga-miR-let7g, gga-miR-1603, gga-miR-1635, gga-miR-1644 and gga-miR-21-5p, whereas gga-miR-1a-3p, gga-miR-1715, gga-miR-196-2, gga-miR-1778 and gga-miR-302b were downregulated (Fig. 5a). In addition, lncRNAs including *AFLP*, *C12ORF43*, *FBXO2* and *LANCL3*, were suppressed by IBDV infection, whereas *MYOZ1*, *TMEM130* and *UBE2QL1* were significantly upregulated (Fig. 5b).

### Establishment of transcription factor–miRNA–mRNA regulatory loops

**Fig. 6:**
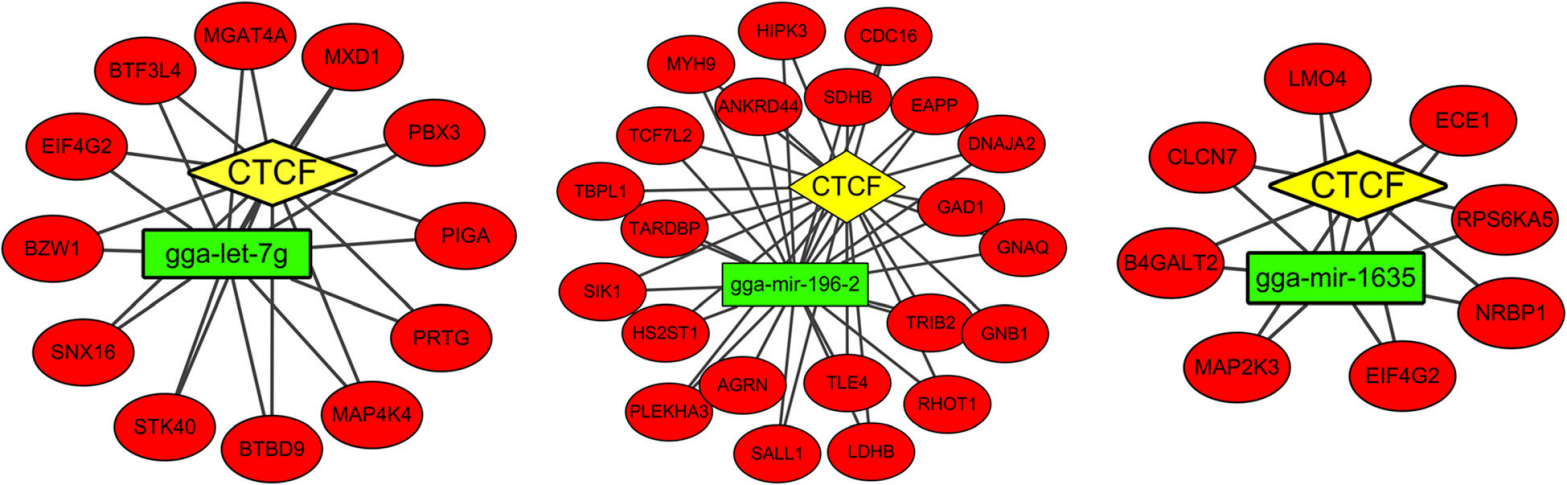
TF–miRNA–mRNA regulatory loops in IBDV-stimulated chicken DCs. A total of 42 TF–miRNA–mRNA interactions involving 1 TF (CTCF), 3 differentially expressed miRNAs (gga-let-7 g, gga-miR-196-2, and gga-miR-1635) regulated by CTCF and 42 differentially expressed mRNAs (also predicted miRNA targets) are summarised for the IBDV-stimulated group. Yellow diamond nodes represent TFs, the green rectangle nodes correspond to miRNAs, and the red ovals represent mRNAs

To obtain a better understanding of the active mechanisms underlying IBDV-infected DCs, TF–miRNA–mRNA regulatory loops were established. First, a total of 165 TF–miRNA combinations were considered, involving 2 TFs and 149 miRNAs (Additional file 10). Considering the differentially expressed miRNAs, we identified three TF–miRNA networks (CTCF-gga-let7g, CTCF-gga-miR-196-2, and CTCF-gga-miR-1635) for IBDV-stimulated DCs (Fig. 6 and Additional file 10). Then, we extracted the CTCF and gene relationship data from ChIPBase. A total of 5830 CFCF–gene pairs were computed and used for constructing TF–miRNA–mRNA regulatory networks by combining the CTCF–gene and miRNA–gene information (Fig. 6 and Additional file 11). For example, *B4GALT2*, *CLCN7*, *ECE1*, *EIF4G2*, *LMO4*, *MAP2K3*, *NRBP1* and *RPS6KA5* could be regulated by CTCF and gga-miR-1635. Surprisingly, we found that *EIF4G2* could be targeted by both gga-miR-1635 and gga-let7g.

## Discussion

DCs play central roles in priming and boosting immune responses [25], and their maturation is pivotal to the development of immunity against many viruses [26]. Viral infection affects the maturation, antigen-presenting ability, and cytokine secretion of DCs [27]. This study showed that difficulties in controlling IBDV infections are related to the immunosuppression of DCs, which would lead to the suppression of T cells [23, 24]. Our previous study revealed that IBDV infection impairs DC maturation and function, which may explain why chickens infected with IBDV fail to trigger effective specific immune responses or develop immune memories. Additionally, the caIBDV could arrest the host’s apoptotic process by inducing apoptotic inhibitors, including NFKBIA/Z, TNFAIP2/3 and ITA, to repress the cell’s immune response. Moreover, the recognition of IBDV is important for cells to activate their surface markers. Raymond [28] found that chicken DF-1 cells may enhance levels of IFIH1, DHX58, and TRIM25, which possess properties for detecting viral dsRNA.

### Total microarray analyses of IBDV-infected DCs

Microarray analyses offer significant insights into the interactions between a virus and its host. Therefore, microarray analyses of DC responses to viral infections could aid in the identification of host defence mechanisms [29]. Our microarray analyses of the alterations in gene expression in IBDV-stimulated DCs provide significant information on specific aspects of molecular pathogenesis and virus–host interactions. Initially, we found that IBDV infection might activate the MAPK and JAK-STAT signalling pathways. In addition, cell migration of DCs might be negatively regulated by IBDV infection, since those differentially expressed genes, induced by IBDV stimulation, were concluded into the negative regulation of cell migration in Fig. 1. The information obtained in this study might provide useful clues for the development of novel preventive or therapeutic strategies against IBDV. Besides direct gene regulation, miRNAs and lncRNAs also affect the functions of DCs with regard to capturing and presenting antigens [16, 19]. In this study, we attempted to determine how miRNAs and lncRNAs induced by IBDV infection regulate the maturation and antigen presentation of chicken DCs. We found that target genes of miRNAs not only influence the MAPK signal pathway, but may also participate in regulating protein localisation and transport, which may block viral migration and replication. Raymond [28] previously demonstrated that upon IBDV infection, regulatory factors including EIF2AK2, MX, GBP7, and IFIT may trigger the IFIT5-IRF1/3-RSAD5 signalling pathway in DF-1 cells, which potentially restricts viral replication during the early infection stage [28]. In addition, we found that the *cis*- and *trans*-targeting of differentially expressed lncRNAs also contributes to the MAPK and JAK-STAT signalling pathways. All of these findings suggest that the MAPK and JAK-STAT signalling pathways are involved in the regulation of chicken DCs stimulated by IBDV.

### The roles of miRNAs and lncRNAs in IBDV infection

Non-coding RNA is a type of RNA involved in regulating processes in defence against viral infection. For example, miRNA, which is small and non-coding, is thought to be a vital and evolutionarily ancient component of genetic regulation. In our study, 991 chicken miRNAs were investigated and 18 differentially expressed miRNAs were identified in IBDV-infected chicken DCs. Of these miRNAs, gga-let-7 g, gga-miR-196-2, gga-miR-1635, gga-miR-1603 and gga-miR-21 were significantly upregulated in IBDV-infected DCs. Previously, gga-miR-21 was suggested to inhibit the replication of IBDV by targeting and suppressing VP1 mRNA translation [30]. This suggests that, in chickens, gga-miR-21 is upregulated as a defence mechanism to fight against IBDV by inhibiting viral replication. Gga-let-7 regulates TGFBR1 and LIN28B during the differentiation process in early chick development [31]. In addition, gga-miR-196, a Hox-related gene, is involved in the regulation of normal development and is mutated in some diseases and malformations [32]. In this context, TFs are involved in the interplay between genes and proteins [12, 13]. In this study, we observed that CREB was involved in IBDV-stimulated DCs (predicted using the KEGG database with the target genes of differentially expressed miRNAs from IBDV-stimulated DCs), indicating that IBDV may activate CREB to facilitate its infection. Conversely, lncRNAs were recently found to play important roles in chicken development and viral infection [33]. Transcriptional regulation by lncRNAs could occur in either a *cis* or a *trans* manner. In our study, we found significant increases in lncRNA (*MYOZ1, TMEM130* and *UBE2QL1*) abundance in IBDV-stimulated DCs. Of these, *TMEM130* may be correlated with animal development [34], whereas *UBE2QL1* was identified as a novel candidate renal tumour suppressor gene [35].

### TF–miRNA–mRNA loops in IBDV-stimulated chicken DCs

TF–miRNA networks were previously identified as important regulatory mechanisms in miRNA regulation [36]. The transcriptional suppressor CTCF is also involved in many cellular processes, including transcriptional regulation, insulator activity, and the regulation of chromatin architecture [37]. Our study recognised three TF–miRNA networks in IBDV-stimulated DCs (CTCF-Let-7 g, CTCF-miR196-2, and CTCF-miR1635). To further explore the mechanisms involved, we studied the TF–miRNA–mRNA loops to determine the mechanisms underlying IBDV infection using ChIPBase, an integrated resource and platform for decoding TF binding sites, expression profiles, and the transcriptional regulation of miRNAs [24]. We identified 42 TF–miRNA–mRNA networks in IBDV-stimulated DCs, which provides new information about the mechanisms underlying the immune responses of chicken DCs to IBDV infection.

## Conclusions

Microarray analyses of chicken DCs stimulated with IBDV provided insight into the mechanisms underlying the host immune response to IBDV infection. Specifically, the MAPK and JAK-STAT signalling pathways were found to be involved in the regulation of chicken DCs stimulated by IBDV. Three TF–miRNA networks were also identified in IBDV-stimulated DCs (CTCF-Let-7 g, CTCF-miR196-2 and CTCF-miR1635). Furthermore, 42 TF–miRNA–mRNA networks were identified in IBDV-stimulated DCs. These data provide valuable insights into host antiviral defence, and supplies useful clues for the development of novel preventive or therapeutic strategies against IBDV.

## Additional files

Additional file 1: Flow cytometry analysis of cell surface molecule expression on avian BMDCs. (More than 70 % of immature avian DCs were CD11c+, while the precentage of MHC Class II+ positive DCs were 64.3 %). (TIF 85 kb)
Additional file 2: qRT-PCR result of the GAPDH and β-actin. (PDF 771 kb)
Additional file 3: Selected data of significant alterated mRNAs stimulated by IBDV (the results of the significantly up- or down-regulated genes were subjecte to *P*-value < 0.05 with a cut off of at least 2-fold change). (XLSX 188 kb)
Additional file 4: Primary GO categorisation, KEGG and BIOCARTA pathway analyses based on differentially expressed mRNAs in IBDV-stimulated chicken DCs. (XLS 111 kb)
Additional file 5: Selected data of significant alterated miRNAs and their target gene predicted by miRDB and miTarget which were stimulated by IBDV (the results of the significantly up- or down-regulated genes were subject to *P*-value < 0.05 with a cut off of at least 2-fold change. Targets genes were predicted using the software miRDB and miTarget, then take into consideration of their intersection with differentially expressed mRNAs). (XLS 2445 kb)
Additional file 6: Primary GO categorisation, KEGG and BIOCARTA pathway analyses (Target gene were predicted by miRDB and miTarget based on differentially expressed mRNAs in IBDV-stimulated chicken DCs). (XLSX 132 kb)
Additional file 7: Selected data of significant alterated lncRNAs stimulated by IBDV (the results of the significantly up- or down-regulated genes were subject to *P*-value < 0.05 with a cut off of at least 2-fold change). (XLSX 32 kb)
Additional file 8: The cis, trans and co-expressed target gene of different expressed lncRNAs. (The cis target genes were selected by mapping the genomic location of lncRNA and those proteins encoding mRNA. The trans target were extracted from the 3′ UTR sequence of different expressed lncRNA and mRNA, which then predicted by RNAplex with the free energy of RNAplex < −100. At last, we calculated the Pearson Correlation Coefficient to predict the lncRNA’s co-expression target genes. The calculation formula is as follows and we selected the target genes according to Pearson correlation coefficient absolute value range of 0.9 and significant correlation with *P* value of 0.01). (XLSX 270 kb)
Additional file 9: Primary GO categorisation, KEGG and BIOCARTA pathway analyses (Target gene (cis, trans and co-expression) of lncRNAs were predicted based on differentially expressed mRNAs in IBDV-stimulated chicken DCs). (XLSX 335 kb)
Additional file 10: Predicted transcription factor (TF )-miRNA regulation loops based on ChIPBase database. (XLSX 415 kb)
Additional file 11: Predicted target genes of miRNA belong to the TF-miRNA loop and TF-mRNA loop. (XLSX 9 kb)

## Abbreviations

3′UTR: Three prime untranslated region
BMDC: Bone marrow dendritic cells
BP: Biological process
BSDC: Bursal secretory dendritic cells
CC: Cellular component
DAVID: The Database for Annotation Visualization and Integrated Discovery
DCs: Dendritic cells
FACS: Fluorescence Activated Cell Sorter
FDC: Follicular dendritic cells
GM-CSF: Granulocyte colony-stimulating factor
GO: Gene ontology
IBDV: Infectious bursal disease virus
JAAS: Jiangsu Academy of Agricultural Sciences
KEGG: Kyoto Encyclopedia of Genes and Genomes
lncRNA: Long non-coding RNA
MAPK: Mitogen-activated protein kinases
MHC-II: Major histocompatibility complex class II
miRNA: microRNA
TDC: Thymic dendritic cells
TF: Transcription factor
