# Supplementary figures and images for: Genome-wide profiling of chicken dendritic cell response to infectious bursal disease
Source: BMC Genomics. 2016 Nov 5;17:878. doi: 10.1186/s12864-016-3157-5 (PMC5097849; doi:10.1186/s12864-016-3157-5)

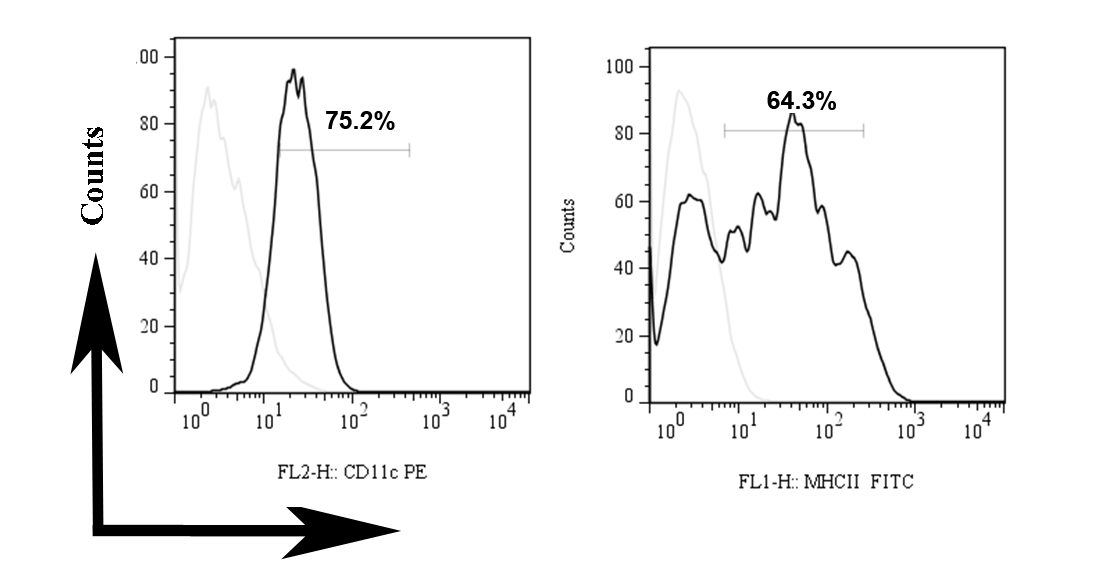

Supplement: Additional file 1: — Flow cytometry analysis of cell surface molecule expression on avian BMDCs. (More than 70 % of immature avian DCs were CD11c+, while the precentage of MHC Class II+ positive DCs were 64.3 %). (TIF 85 kb) [file 12864_2016_3157_MOESM1_ESM.tif]
