# Supplementary material for: Genome-wide profiling of chicken dendritic cell response to infectious bursal disease
Source: BMC Genomics. 2016 Nov 5;17:878. doi: 10.1186/s12864-016-3157-5 (PMC5097849; doi:10.1186/s12864-016-3157-5)
Supplement: Additional file 2: — qRT-PCR result of the GAPDH and β-actin. (PDF 771 kb) [file 12864_2016_3157_MOESM2_ESM.pdf]

Additional file 2. qRT-PCR result of the GAPDH and β-actin

Sample Name	Ct（GAPDH）	Sample Name	Ct（β -actin）
blank	25.16869	blank	20.84561
blank	23.20909	blank	20.83502
blank	23.4249	blank	20.69427
blank	23.00589	blank	20.82916
IBDV infection	24.13549	IBDV infection	20.45815
IBDV infection	23.47066	IBDV infection	20.52028
IBDV infection	22.89913	IBDV infection	20.82106
IBDV infection	23.2759	IBDV infection	20.63129
